# Morphology Of Current Of Injury Does Not Predict Long Term Active Fixation ICD Lead Performance

**Published:** 2009-03-15

**Authors:** Hanno Oswald, Benjamin Husemann, Ajmal Gardiwal, Christoph Lissel, Maximilian A Pichlmaier, Ulrich Luesebrink, Thorben Koenig, Gunnar Klein

**Affiliations:** 1 Department of Cardiovascular Medicine, Hannover Medical School, Hannover, Germany; 2 Department of Thoracic and Cardiovascular Surgery, Hannover Medical School, Hannover, Germany

**Keywords:** ICD, lead, current of injury, endocardial unipolar electrogram

## Abstract

**Background:**

Currents of injury (COI) have been associated with improved lead performance during perioperative measurements in pacemaker and ICD implants. Their relevance on long term lead stability remains unclear.

**Methods:**

Unipolar signals were recorded immediately after active fixation ICD lead positioning, blinded to the implanting surgeon.  Signals were assigned to prespecified COI types by two independent investigators. Sensing, pacing as well as changes requiring surgical intervention were prospectively investigated for 3 months.

**Results:**

105 consecutive ICD lead implants were studied. All could be assigned to a particular COI with 48 type 1, 43 type 2 and 14 type 3 signals.  Pacing impedance at implant was 703.8±151.6 Ohm with a significant COI independent drop within the first week. Sensing was 10.6mV± 3.7mV and pacing threshold at implant was 0.8±0.3mV at 0.5ms at implant. There was no significant difference between COI groups at implant and during a 3 months follow up regarding sensing, pacing nor surgical revisions.

**Conclusions:**

Three distinct patterns of unipolar endocardial potentials were observed in active fixation ICD lead implant, but COI morphology did not predict lead performance after 3 months.

## Introduction

 ICD implantation using transvenous leads has been shown to cause endomyocardial injury detectable in blood tests for cardiomyocyte damage [1]. Currents of injury (COI) have been described for pacemaker and ICD lead implants of both active and passive fixation techniques with ST segment elevation on endocardial electrogram tracings. It has been shown, that ST segment elevations of 2mV and more are associated with beneficial endocardial lead stability, less frequent lead dislogements and beneficial sensing and pacing capabilities at the cost of slightly higher rates of myocardial perforations during short term perioperative measurements [2-4]. Currents of injury can be detected immediately after lead fixation with spontaneous resolution after approximately 10 minutes [4].

Currently, there is only rare prospective data regarding the clinical relevance of COI as a predictor of long-term endocardial lead performance, defined as the first three months after lead implantation [2]. Furthermore, it has been speculated, whether morphology of COI on bipolar tracings might represent a useful tool for prediction of lead stability for lead implantation [3,5]. Varriale et al. hypothesized, that a negative unipolar COI might be a sign of right ventricular ischemia and consequently not ideal for lead placement. Thus, this prospective single blinded study in a single type active fixation mechanism ICD lead describes 1) the distribution of different COI morphology patterns in an unselected group of ICD implantations and investigates the role of the morphology of currents of injury for 2) its impact on sensing and pacing capabilities and 3) its clinical relevance on lead dislogements and surgical interventions during the first three months after lead implantation.

## Methods

### ICD implantation

Patients receiving standard transvenous ICD lead implant of a prespecified single type active fixation mechanism (Sprint Fidelis 6931 and 6949 lead, Medtronic, Minneapolis, Minnesota, USA) were eligible for this study. ICD device selection was not restricted to any manufacturer or type. The study was approved by our local board of ethics. Briefly, following preparation of a left sided subfascial prepectoral or subpectoral ICD pocket, central venous access was achieved by subclavian vein puncture using Seldinger technique. Once the electrode was positioned in a stable right ventricular apex position, the COI was recorded using the analyzer ERA 300 (Biotronik, Berlin, Germany) with unipolar connection to the distal electrode of the ICD lead at a paper speed of 25mm/s and amplifier gain of 2mm/mV immediately after screw deployment. The implanting surgeon received values for sensing amplitude, pacing threshold and pacing impedance. He was not aware of the printed COI. Finally, every lead was fixated at its suture-sleeve to the pectorals fascia using non absorbable sutures, and all measurements were repeated through ICD telemetry. These final values were considered 'implant values' for further evaluation. Testing for detection and successful shock therapy of ventricular fibrillation at 10 Joule below maximum shock energy was performed in all cases.

### Definition of currents of injury

**Figure 1:**
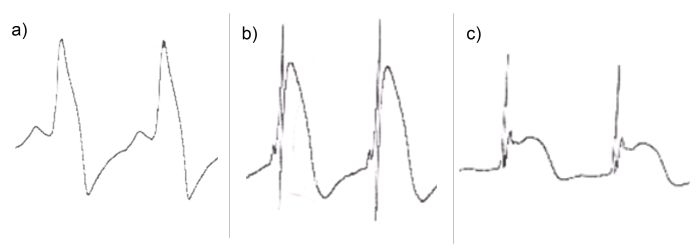
Representative examples of type of COI are shown. a) Type 1 potential b) type 2 potential and c) type 3 potential. Recorded at paper speed of 25mm/s, 2mm/mV. Examples were amplified individually for display.

Three different patterns of endocardial unipolar potentials following transvenous active fixation lead positioning were observed during previous routine ICD lead implants. They were observed to be present temporarily for less than 10 minutes following lead positioning before resolving into a narrow chronic R-spike signal. These distinct types of COI were therefore prespecified as shown in Figure 1.

A type 1 potential showed a markedly widened positive R spike with rapid upslope and a shoulder before downslope to negative values than baseline with broad normalisation to baseline thereafter. A type 2 potential was characterized by a sharp positive spike and a second plump positive wave, which was set apart from the sharp first spike. The type 3 potential also had a sharp first spike with a second broad saddle back type plateau phase without a negative later part.

### ICD follow up

Measurements for sensing amplitude, pacing impedance and pacing threshold were performed before hospital discharge within five days after implant, at one month and at three months following ICD implant in our outpatient clinic. Chest X-ray was performed to confirm ICD lead position prior to hospital discharge and at further follow up, whenever relevant changes in sensing and pacing measurements were noticed at investigators discretion. Changes in sensing and pacing capabilities were assumed 'severe', when later surgical interventions were required for lead repositioning during follow up.

### Measurements

Right ventricular pacing impedance, pacing threshold and sensing amplitudes were measured within each ICD at the end of the implant procedure, within one week at hospital discharge and after three months. Relative values for sensing amplitudes were calculated with the implant value being 100% for comparison during follow up due to an expected large interindividual spread of sensing amplitudes. Since some ICD manufacturers used pulse width testing and others amplitude testing for pacing thresholds, thresholds during follow up were standardized as pacing impulse energy [amplitude (Volt) squared multiplied by pulse width (ms)] for quantitative comparisons during follow up [5]. Baseline threshold energy was subtracted from threshold energy at three months after implant in order to demonstrated changes in pacing thresholds over time. For qualitative assessment of clinical relevance of changes in pacing thresholds during follow up, thresholds were classified as 'of unchanged or better' versus 'worse' for each patient looking at pacing amplitudes at fixed pulse widths in generators with amplitude threshold testing or pulse width at identical amplitudes in devices with pulse width tests.

### Statistics

Data are presented as mean ± standard deviation (SD). For data with great interindividual variations such as sensing amplitudes, relative values are calculated with implant values being 100%, thus later follow up values were compared to implant values in percent. Statistical analyses were calculated using the SPSS 14.0 statistics software (SPSS, Chicago, Illinois). Groups were compared by unpaired Chi-Square test. A p<0.05 was considered statistical significant.

## Results

### Patient baseline characteristics and currents of injury

105 consecutive patients received ICD lead implants at our institution from November 2005 until December 2006 and were eligible for study inclusion. Patient baseline characteristics are shown in Table 1.

During local anaesthesia with no or only mild sedation, 101 ICD (96.8%) were implanted in a prepectoral pocket and 4 patients (3.8%) received a subpectoral device implant. Our overall population showed congestive heart failure symptoms with a mean NYHA class of 2.1 with a narrow QRS width of 112.5±32.2ms.  85% of the population was male with 69.5% ischemic cardioymyopathy and 69% secondary prophylactic indication for ICD therapy.

All patients were assigned to one of the three prespecified types of COI potential by two independent investigators on visual assessment. There were no disagreeing classifications for any COI studied.

48 patients were assigned to the type 1 potential group, 43 patients were assigned to the type 2 group and 14 patients were assigned to the type 3 potential group. Baseline criteria were equally distributed among the three types of COI.

### Sensing

**Figure 2:**
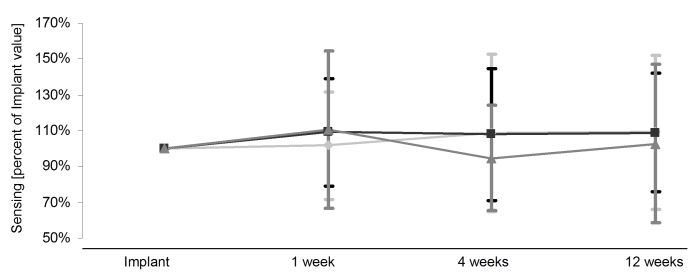
Right ventricular sensing during the three months follow up. Relative values are shown with the implant value being 100%. Light grey diamonds (♦) represent type 1 potentials, black squares (■) represent the group of type 2 potentials and grey triangles (▲) represent the group of type 3 potentials. Data presented as mean ± SD.

Appropriate sensing amplitudes were achieved in all patients with overall mean R spike amplitude of 10.6mV± 3.7mV measured through ICD telemetry at implant with no differences between groups of currents of injury. All patients had signals greater than 5mV. Given a wide spread of inter-individual right ventricular sensing amplitudes, relative values were calculated with the implant value being 100% for comparison during follow up as shown in Figure 2. An increase of right ventricular amplitudes compared to implant values was noticed in all three groups within the three months follow up, while those with type 1 and type 2 had slightly but not statistically significantly higher sensing amplitudes than patients with a type 3 potential.

### Pacing impedance

**Figure 3:**
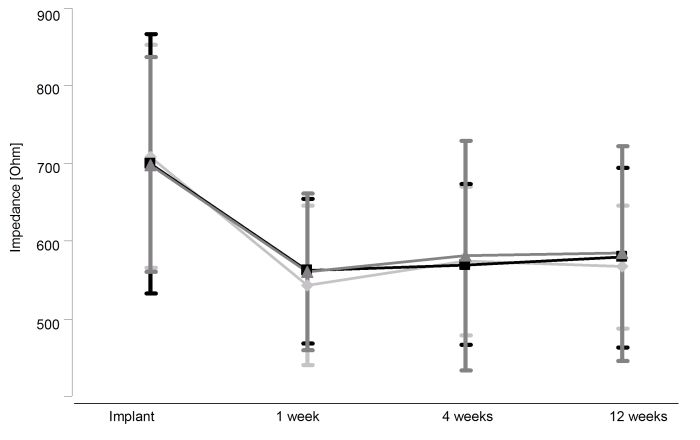
Pacing impedance in Ohm during the three months follow up. Light grey diamonds (♦) represent type 1 potentials, black squares (■) represent the group of type 2 potentials and grey triangles (▲) represent the group of type 3 potentials. Data presented as mean ± SD.

Mean pacing impedance measured through ICD telemetry at the end of ICD lead implant was 703.8±151.6 Ohm with no inter-group difference. There was a significant (p<0.05) decrease in pacing impedance within the first week after implant for all COI groups with no significant difference depending on the type of potentials of injury as shown in Figure 3. Impedance remained unchanged during further follow up after the first week drop.

### Pacing threshold

Appropriate pacing thresholds were achieved in all patients with analyzer based mean unipolar threshold 0.8±0.3V at a preset pulse width of 0.5ms. Qualitative development of right ventricular pacing at three months following lead implantation is shown in Table 2.

**Figure 4:**
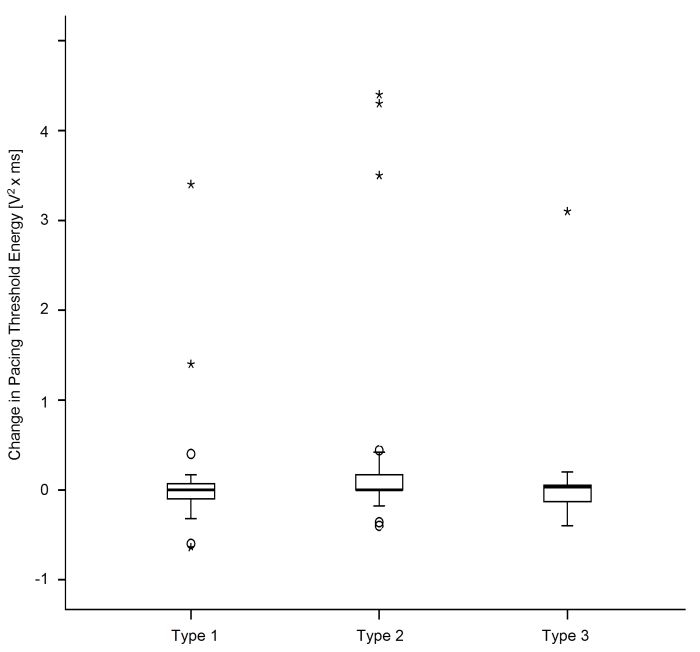
Pacing threshold energy change calculated by subtracting baseline values from the pacing threshold energy at three months follow up. An increase greater than '0' therefore represents an increase in pacing threshold energy (impaired threshold) after three months while values less than '0' describe a decreased energy (improved threshold). Data presented as whiskerblot. The box represents percentile 25 through 75 with each group median marked by inner lines and group ranges with minimal and maximum values between bars.

Changes in pacing threshold energy between implant and three months follow up are presented in Figure 4. There were no significant differences for neither qualitative nor quantitative comparisons of pacing threshold between COI groups at three months after lead implant.

### Drop outs prior to three months follow up

Six patients did not complete the three months follow up. There were five deaths reported within the three months follow up period with 60% cardiac and 40% non cardiac mortality and one drop out for heart transplant. One patient died of previously unknown bronchial carcinoma and one patient died of mechanical intestinal obstruction. There were three deaths for refractory congestive heart failure. None of the above deaths were judged to be ICD or implant related. Three patients did not undergo pacing threshold testing at three months follow up, one in each COI group.

### Surgical revisions for lead complications

Six patients received second surgery for a lead related complication. Five patients had micro dislodgement defined as loss of capture or loss of sensing and one patient had surgical revision for right ventricular perforation with pericardial effusion without further complications. No patient with a type 3 COI received a second operation while four were revised in type 1 (three micro dislodgements, one perforation) and two for micro dislodgement in the type 2 group. No macro dislodgements of ICD leads as apparent on chest radiograph were documented in any of the above patients. Morphology of COI did not predict later need for revision.

## Discussion

Our single-blinded single-center study addressed the role of COI on ICD lead performance in a single type active fixation mechanism cohort. Morphology of COI did not predict alterations of sensing and pacing capabilities as well as the need for surgical lead revisions during a follow up of three months. With mostly male patients with impaired left ventricular function due to ischemic cardioymyopathy and secondary prevention ICD indication of ventricular tachyarrhythmia, a standard cohort for current ICD recipients was investigated (6). Results for morbidity and mortality were comparable to previously published typical ICD cohorts [7,8].

Six surgical interventions for lead complications were reported during our follow up of 3 months. None were in the type 3 potential group, four in the type 1 potential group with 3 micro dislodgements and one perforation, while 2 micro dislodgements were reported for the type 2 potential group. This trend towards more complications in type 1 and type 2 potentials was not statistically significant. Sensing amplitudes, pacing impedance and pacing threshold was not statistically significantly affected by the type of endocardial potential at implant. A significant decrease of pacing impedance was observed independent of the COI within the first week after implant as previously described [10]. Pacing threshold development was heterogeneously distributed during follow up independent of the type of endocardial potential at implant. No episodes of oversensing or short RR cycle lengths were noticed in our cohort within the three months follow up period in regard to previously published early lead failure [9].

Previous single-center studies have found a beneficial effect on short term lead stability for significant currents of injury in perioperative investigations, mostly reporting results during the first 24 hours after lead implantation. Both, active and passive fixation leads were suggested to be more stable at the end of the implant procedure, when significant COI were quantitatively measured during lead positioning [3,4].

Since it has been speculated, that apart from ST segment elevation and duration of COI, morphology of COI might be a predictor of lead stability [3,10], we assessed three distinct patterns of COI. Three distinct patterns of unipolar endocardial signals were observed during earlier routine lead implants as presented in  Figure 1, showing differences in upstroke at the onset of the potential and also negative deflections at the end of the potential. It has been hypothesized from earlier reports, that negative COIs are an indicator of local ischemic injury and thus leads implanted at this area are associated with good fixation but poor pacing thresholds [3]. We chose to investigate unipolar recordings immediately after screw deployment as previously described [10]) in order to see only effects from the very distal tip of the ICD lead including its screw. Bipolar recordings on the other hand have been described for short term correlations to lead performance by Redfearn et al. and Saxonhouse et al., which may limit comparability to both previous studies [3,4], but we believe, that COI are mostly, if not exclusively, caused by endocardial injury at the distal electrode tip and screw. Since we did not investigate the effect of COI on immediate but on long-term lead performance at three months, comparisons with previous studies by Saxonhouse et al. and Redfearn et al. are difficult. Due to the single-blinded design of our study with the implanting surgeon being unaware of the COI, ICD implant was continued following successful lead placement. No short term developments of COI morphology were recorded. Changes of transient COI morphology between the previously described prespecified groups are possible even though recordings were performed immediately after active fixation.

Notably, our study focuses on active fixation ICD leads, whereas other investigated pacemaker leads only (Redfearn) or a mixed pacemaker and ICD population (Saxonhouse). In our study, only one active fixation mechanism of the Sprint Fidelis lead (6931 and 6949) was used. Different design of ICD leads compared to pacemaker leads might be one reason, why results of earlier pacemaker studies can not be translated into ICD lead recipients. The stiffer design of ICD leads might influence their behaviour concerning macro dislogements. Different indications for lead implantation regarding lead performance might also influence our results, because pacing characteristics are of minor importance in most ICD recipients compared with pacemaker patients. However, the opposite might be true for sensing capabilities.

Our study demonstrates that COI morphology cannot be used to predict long term lead performance. Short term perioperative observations by Redfearn et al. and Saxonhouse et al. on the other hand are not rebuted by our study, but we extend their results of quantitative COI with qualitative assessment during a longer period of three months.

## Conclusions

Quantitative COI measurement might predict short term ICD lead performance. However, qualitative morphologic assessment of COI does not add prognostic information for long-term (three months) ICD active fixation lead performance.

## Study limitation

Since the distribution of different COI morphology patterns in an unselected group of ICD screw in lead implantations was unknown at the time the study was performed, our study may be underpowered to prove the hypothesis, that a particular type of COI  is superior in predicting long term lead performance. However, our study is the first to describe the distribution of COI morphology patterns in this setting systematically. The number of cases included in this study is large compared to published data on this topic. A larger trial with sample size calculation based on our data may help to further prove the hypothesis, that COI morphology does not predict ICD active fixation lead performance.

## Figures and Tables

**Table 1:**
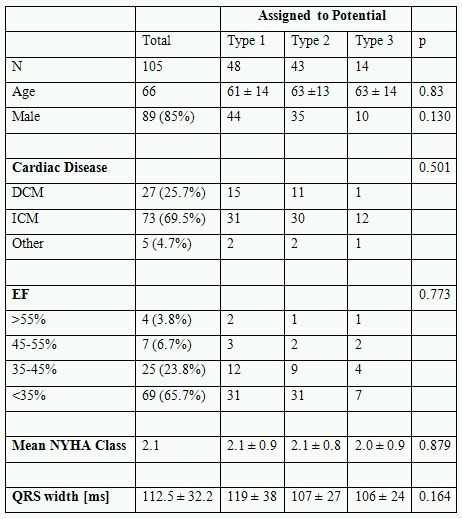
Baseline characteristics of the total cohort of 105 lead implants as well as subgroup characteristics depending on the type if endocardial signal at implant.

DCM: Dilative Cardioymyopathy. ICM: Ischemic Cardioymyopathy. EF: Ejection Fraction. Data presented in mean ± SD.

**Table 2:**
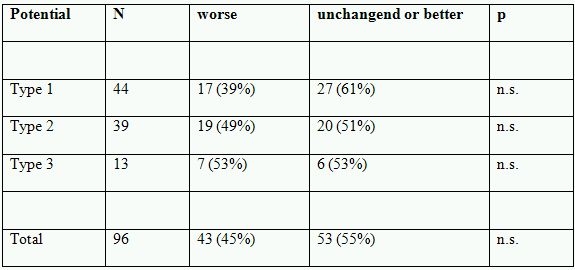
Pacing threshold at three months follow up qualitatively classified as 'unchanged or better' or 'worse' compared to implant measurements within each group of COI.
